# Evaluation of Alignment between the Health Claims Nutrient Profiling Scoring Criterion (NPSC) and the Health Star Rating (HSR) Nutrient Profiling Models

**DOI:** 10.3390/nu10081065

**Published:** 2018-08-10

**Authors:** Elizabeth K. Dunford, Liping Huang, Sanne A. E. Peters, Michelle Crino, Bruce C. Neal, Cliona Ni Mhurchu

**Affiliations:** 1 The George Institute for Global Health, University of New South Wales, Sydney, NSW 2052, Australia; edunford@georgeinstitute.org.au (E.K.D.); hliping@georgeinstitute.org.cn (L.H.); mcrino@georgeinstitute.org.au (M.C.); bneal@georgeinstitute.org.au (B.C.N.); 2 Faculty of Medicine, University of Sydney, Sydney, NSW 2052, Australia; 3 The George Institute for Global Health, University of Oxford, Oxford, OX1 2BQ, UK; sanne.peters@georgeinstitute.ox.ac.uk; 4 Department of Epidemiology and Biostatistics, School of Public Health, Faculty of Medicine, Imperial College London, London SW7 2AZ, UK; 5 National Institute for Health Innovation, University of Auckland, Auckland 1142, New Zealand

**Keywords:** food composition, nutrient profiling, food labelling, Health Star Rating, health claims

## Abstract

In Australia, manufacturers can use two government-endorsed approaches to advertise product healthiness: the Health Star Rating (HSR) front-of-pack nutrition labelling system, and health claims. Related, but different, algorithms determine the star rating of a product (the HSR algorithm) and eligibility to display claims (the Nutrient Profiling Scoring Criterion (NPSC) algorithm). The objective of this study was to examine the agreement between the HSR and NPSC algorithms. Food composition information for 41,297 packaged products was extracted from The George Institute’s FoodSwitch database. HSR and the NPSC scores were calculated, and the proportion of products in each HSR category that were eligible to display a health claim under the NPSC was examined. The highest agreement between the HSR scoring algorithm and the NPSC threshold to determine eligibility to display a health claim was at the HSR cut-off of 3.5 stars (*k* = 0.83). Overall, 97.3% (*n* = 40,167) of products with star ratings of 3.5 or higher were also eligible to display a health claim, and 94.3% (*n* = 38,939) of products with star ratings less than 3.5 were ineligible to display a health claim. The food group with greatest divergence was “edible oils”, with 45% products (*n* = 342) with HSR >3.5, but 64% (*n* = 495) eligible to display a claim. Categories with large absolute numbers of products with HSR <3.5, but eligible to display a claim, were “yoghurts and yoghurt drinks” (335 products, 25.4%) and “soft drinks” (299 products, 29.7%). Categories with a large number of products with HSR ≥3.5, but ineligible to display a claim, were “milk” (260 products, 21.2%) and “nuts and seeds” (173 products, 19.7%). We conclude that there is good agreement between the HSR and the NPSC systems overall, but divergence in some food groups is likely to result in confusion for consumers, particularly where foods with low HSRs are eligible to display a health claim. The alignment of the NPSC and HSR scoring algorithms should be improved.

## 1. Introduction

Diet-related diseases, including obesity, type 2 diabetes, and cardiovascular diseases, are increasing globally in conjunction with greater consumption of processed foods [1]. Processed foods contribute more than two thirds of dietary energy and nutrients consumed in high-income countries [2], indicating that processed foods have substantial potential to influence the healthiness of population dietary intake. Common recommendations to improve population diets include implementation of effective front-of-pack nutrition labelling systems, application of food and beverage taxes on unhealthy products, and restrictions on marketing of unhealthy foods to children [3]. Nutrient profiling, defined as “the science of classifying or ranking foods according to their nutritional composition for reasons related to preventing disease and promoting health” [4], is often used to underpin such policies [5].

**Figure 1:**
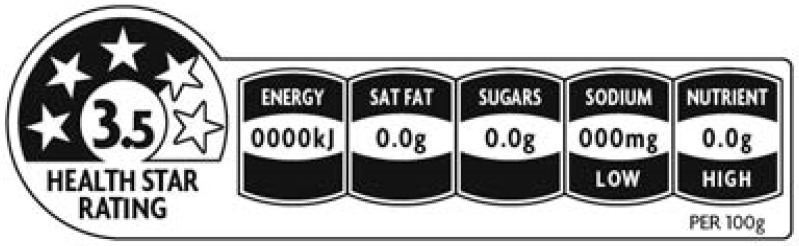
Example of the Health Star Rating front-of-pack logo.

In Australia, manufacturers can advertise the healthiness of their product offerings in a number of ways. Two government-endorsed systems are regulated health claims on product packaging, and voluntary front-of-pack nutrition labels. The Nutrient Profiling Scoring Criterion (NPSC) was developed by Food Standards Australia New Zealand (FSANZ) to determine whether or not a product is eligible to display a health claim [6]. The NPSC algorithm assigns a score based on the overall nutritional content of a product. Based on the score received, a product is deemed to either meet the criteria to make a health claim or not. In 2014, the Australian and New Zealand governments endorsed the use of a voluntary interpretive front-of-pack labelling system, the Health Star Rating (HSR), for packaged foods [7]. The HSR system aims to make it easier for consumers to identify healthier products in the same category, and to be consistent with the 2013 Australian Dietary Guidelines. The HSR algorithm assigns a product a rating between half a star and five stars, in half-star increments, with a higher number of stars indicating a healthier product (Figure 1).

The scoring algorithms that underpin both the NPSC and HSR originate from the UK Food Standards Agency Nutrient Profiling Model 2004/5 (UK NPM), which was developed as a tool to determine which foods are permitted to be advertised during children’s television programming [8]. All three models score foods based on their content of four “negative” nutrients i.e. energy, saturated fat, total sugars, sodium, and three “positive” components, fibre, protein, and fruit, vegetable, nut and legume content (FVNL). The UK NPM divides products into two categories (foods and non-alcoholic drinks) and scores are awarded to products based on dietary reference values (DRV). The score bands for negative (baseline) nutrients start at 3.75% of the DRV, with subsequent intervals of 3.75% of the DRV; thus, the maximum points for negative nutrients (10 points) are equivalent to 37.5% DRV. The score bands for positive food components also start at 3.75% DRV, with subsequent intervals of 3.75% DRV. The maximum scores for positive components are capped at five points (equivalent to 18.75% DRV).

In 2013, FSANZ introduced a standard for nutrition and health claims on food labels and in food advertisements, and the NPSC was developed to determine if a food was suitable to make a health claim. The NPSC was adapted from the UK NPM with relatively little modification, the main difference being the addition of a third category for oils, spreads, and cheese (foods with a low saturated fat content relative to total fatty acid content). Baseline points for foods in this category (Category 3) were extended linearly to 11 points for energy, 30 points for saturated fat, and 30 points for sodium. The scoring scales for baseline nutrients for other foods and beverages (Categories 1 and 2) remained unchanged, i.e., maximum 10 points.

Lesser modifications for NPSC included: (1) the start point for total sugar scoring was increased from 4.5 g/100 g to 5 g/100 g, to ensure that plain milks were eligible to display health claims; (2) the scoring scale for FVNL was extended from five to eight points, to ensure that nuts could carry health claims; (3) potatoes contribute to FVNL content, reflecting their categorization as vegetables in Australian and New Zealand dietary guidelines, although they (and other starchy vegetables) are excluded from FVNL in the UK model; and (4) the eligibility cap to score protein points was increased from <11 to <13, to ensure some breakfast cereals could carry claims.

The HSR nutrient profiling model was based on the NPSC system, and intended to align with it, but there are notable differences between the systems: (1) in HSR, products are assigned to one of six categories because the three original NPSC categories were each subdivided into dairy and non-dairy foods/beverages; (2) although the NPSC scoring for Category 3 foods (dairy and non-dairy) was maintained, scoring scales for baseline nutrients for Category 1 and 2 foods/beverages were extended to 11 points for energy, 30 points for saturated fat, 22 points for total sugars, and 30 points for sodium; (3) the scoring scale for FVNL was expanded from 5 to 8 points, whilst those for fibre and protein were expanded from 5 to 15 points. Assignment of a star rating from the final HSR score was based on score distributions in a database of approximately 3000 Australian foods. For non-dairy foods, the 1st and 99th percentile scores were selected as cut-offs for the “most healthy” (5 stars) and “least healthy” (½ star) endpoints, respectively. For dairy foods, the 5th and 99th percentile scores were used.

Around 14% of Australian food and beverage products display a health or nutrient content claim [9], and more than 3500 food and beverage products display an HSR on-pack [10]. The objective of this study was to evaluate the agreement between these two schemes, which both aim to support and promote healthier food and beverage choices for Australian consumers, but use different scoring algorithms.

## 2. Materials and Methods

### 2.1. Data Sources

The George Institute for Global Health’s FoodSwitch Database was used for this project. Methods have been described in detail elsewhere [11], but in brief, the full FoodSwitch database contains annually updated nutritional information for packaged food products collected from four major Australian supermarkets supplemented by product data provided direct by manufacturers and crowdsourced through the FoodSwitch smartphone application. The database represents more than 90% of the Australian packaged food market [12]. The following fields of information from mandatory back-of-pack Nutrition Information Panels (NIP) were extracted for use in this analysis: product description, energy (kJ/100 g), protein (g/100 g), saturated fat (g/100 g), total sugars (g/100 g), sodium (mg/100 g), fibre (g/100 g, where recorded), and FVNL (%, where recorded). FVNL values were not reported for some products, in which case, estimates were made using information from the product ingredient lists and/or known values for similar food products. Fibre content was, likewise, not reported on the NIP for some products, and a comparable estimation method was used. Methods for FVNL and fibre estimations have been published previously [11].

### 2.2. Food Categories Included

Foods were classified into major groups (e.g., bread and bakery products), categories (e.g., bread), and subcategories (e.g., pita bread) based on the system established by the Global Food Monitoring Group, which is designed to enable monitoring of the healthiness of national packaged food supplies [13]. Excluded from the current analysis were alcohol, dried herbs and spices, salt, vitamins and supplements, and bottled water, as these products are not required to display a NIP according to the Australia New Zealand Food Standards Code. Fresh and frozen fruit and vegetables without nutritional information were also excluded. Fitness products, and baby and infant foods, were excluded, as they are not permitted to display a HSR on-pack, and infant formulae are not permitted to display a health claim on-pack. In addition, single ingredient foods (e.g., eggs, honey, sugar), variety packs, and products that were missing nutrient information essential for calculating HSR and NPSC scores, were excluded.

### 2.3. Calculation of the HSR

Calculation of the HSR was performed using criteria specified in the “Guide to the Health Star Rating Calculator”, endorsed by the Australian government [14]. Allocation of all packaged products in the FoodSwitch database to one of the six HSR categories was determined on the basis of product name and ingredients. The HSR was calculated by (1) assigning baseline points for energy, saturated fat, total sugar, and sodium content per 100 g; (2) awarding modifying points for FVNL content, protein, and fibre where applicable; (3) calculating an overall score by subtracting modifying points from baseline points, with a lower score reflecting a more nutritious food product; and (4) assigning a HSR (from 0.5 to 5.0 stars in half-star increments) according to the overall score using the defined scoring matrix. Figure S1 (A) outlines an example of an HSR calculation.

### 2.4. Calculation of the NPSC

Calculation of the NPSC was performed using criteria specified by the Australian government [6]. Allocation of all packaged products in the FoodSwitch database to one of the three NPSC categories was determined on the basis of product name and ingredients. The NPSC was calculated by (1) assigning baseline points for energy, saturated fat, total sugar, and sodium content per 100 g; (2) awarding modifying points for FVNL content, protein, and fibre where applicable; (3) calculating an overall score by subtracting modifying points from baseline points, with a lower score reflecting a more nutritious food product. For Category 1 (beverages), products with a score <1 are eligible to display a health claim. For Category 2 (foods), products with a score <4 are eligible, and for Category 3 (oils, etc.), products with a score <28 are eligible. Figure S1 (B) outlines an example of an NPSC calculation.

### 2.5. Statistical Analysis

The mean and standard deviation (SD) HSR were calculated overall, and by food category. The proportion of products eligible to display a health claim using the NPSC was also determined overall and for each major food category. Graphical representations of the data were inspected for comparability of distribution of HSRs, HSR scores, and NPSCs. To explore agreement between the HSR and NPSC in relation to how the two schemes would appear on food packages, agreement between the NPSC and HSR at each HSR value (star rating) was determined using Cohen’s Kappa. The ability of the HSR to discriminate between products that are and are not eligible to carry a health claim was determined using the area under the receiver operating characteristic curve (AUC). The sensitivity and specificity were calculated for each HSR value. All analyses were performed using Stata v15.1 (StataCorp LLC, College Station, TX, USA).

## 3. Results

The FoodSwitch database contained information on 47,116 products, of which 41,297 were included in these analyses. Another 5819 (12.4%) products were excluded for one of the following reasons: not required to display a NIP (*n* = 970), in an excluded category (*n* = 2110), or data on an essential nutrient required for the calculation of HSR or NPSC scores (i.e., energy, saturated fat, total sugar, sodium, protein) were missing (*n* = 2739). The number of products in each major food category ranged from 318 in “foods for special dietary use” to 5515 in “dairy”.

### 3.1. HSR of Packaged Foods

The mean HSR for all 41,297 packaged products was 2.8 stars (SD 1.4). Forty-five percent of products received 3.5 stars or more. Food categories with the highest proportion of products eligible for 3.5 stars or more were “seafood” (82%) and “foods for special dietary use” (70%). The categories with the lowest proportion of products, eligible for 3.5 stars or more, were “confectionery” (4.8%) and “bread and bakery products” (28.7%) (Table 1). The most frequent star rating achieved was 4.0 (15% products), and 4.5 stars was the least frequent (7%) (Table 2). The distribution of HSR scores varied by food category, but aside from “dairy” and “non-alcoholic beverages”, most had approximately normal distributions (Figure S2).

### 3.2. NPSC of Packaged Foods

Forty-eight percent of all products were eligible to display a health claim (Table 1). Categories with the highest proportion of products eligible to display a health claim were “convenience foods” (78%) and “seafood” (74%), and those with the lowest proportion of products eligible to display a health claim were “confectionery” (<7%) and “bread and bakery products” (30%) (Table 1). The distribution of the NPSC scores varied by food category, but except for “cereal and cereal products”, “dairy”, and “non-alcoholic beverages”, distributions were approximately normal (Figure S3).

### 3.3. Agreement between HSR and NPSC

**Figure 2:**
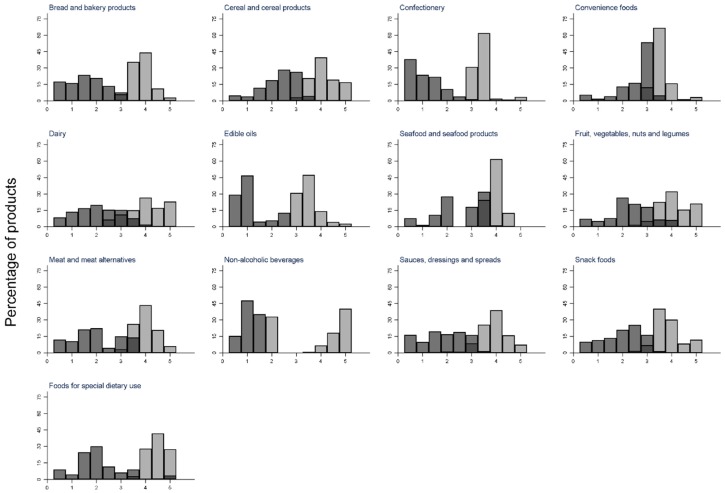
Health Star Rating distribution of products eligible (light grey) and ineligible (medium grey) to display a health claim, by major food category. Dark grey indicates overlap between products eligible and ineligible to display a health claim.

The AUC for discrimination, between products that are and are not eligible to carry a health claim by the HSR, was 0.963. The highest agreement between the HSR scoring algorithm and the NPSC threshold, to determine eligibility to display a health claim, was at the HSR cut-off of 3.5 stars (*k* = 0.83) (Table 2). At this cut-off, the sensitivity was 88%, and the specificity was 95%. There were only 2.7% of products that received ≥3.5 stars that were ineligible to carry a health claim under the NPSC (Table S1 and Figure S4), and only 5.7% of products with a HSR of less than 3.5 were eligible to carry a health claim. Better agreement was seen at very low and very high HSRs with less than 1% of products with star ratings of 0.5, 1.0, and 1.5 stars eligible to display a health claim, and >99% of products with star ratings of 4.5 and 5.0 stars eligible to display a health claim (Table 2 and Figure 2).

The food group with the greatest proportion of products in alignment was “bread and bakery products”, with only 1.8% of products with an HSR of <3.5 eligible to display a health claim under the NPSC, and only 0.3% of products with HSR ≥3.5 ineligible to display a health claim. The group with the largest proportion of foods in divergence was “edible oils” (Figure S4), with 19.9% of products (*n* = 153) with an HSR of <3.5 deemed to be eligible to display a health claim (Table S1). The divergence between the two schemes was apparent (>15%) in all subcategories in this group (“edible oils”, “cooking oils”, and “oil spray”) except coconut oil. Similarly, 19.5% of products in the “non-alcoholic beverage” category (*n* = 678) had an HSR of <3.5, but were eligible to display a health claim, with divergence most apparent within the “flavoured water” subcategory (Table S1 and Figure S4).

The categories with most divergence in terms of the absolute number of products with HSR <3.5, but eligible to display a health claim, were “yoghurts and yoghurt drinks” (339 products), “soft drinks” (299 products), and “packaged fruit” (179 products). The categories with most divergence in terms of the absolute number of products with HSR ≥3.5 but ineligible to display a health claim were “milk” (260 products), “processed meat” (238 products), and “nuts and seeds” (173 products).

## 4. Discussion

The HSR and health claims systems are generally well aligned in terms of allocated star ratings and eligibility to display health claims. However, there are some notable anomalies. Approximately 6% of products (*n* = 2358) were eligible to display a health claim despite scoring a HSR <3.5 stars. Conversely, 2.7% (*n* = 1130) were ineligible to display a health claim, despite rating ≥3.5 stars. Such divergence in health claims and the HSR could result in confusion amongst consumers, because although the proportion of products affected by such discrepancies is relatively small, it translates into a large absolute number of products (*n* = 3488). Given the known difficulties that consumers already have with interpreting nutrition labelling, there is a strong case for ensuring better alignment between the two Australian government-endorsed systems to guide healthier food choices, especially for food categories where the alignment is most problematic (oils, flavoured waters, and yoghurts).

One reason for divergence between the two systems may be non-alignment of NPSC threshold scores used to determine if foods are eligible to display health claim and a HSR rating of 3.5 stars. For example, in “edible oils” and “non-alcoholic beverages”, the threshold scores to determine eligibility to display a health claim are 28 and 1 respectively, but a HSR of 3.5 corresponds to lower (healthier) scores of 23 and −3. Another example is “milk products”, where 21% were ineligible to display a health claim, although their HSR was ≥3.5. The discrepancy may be because NPSC determines that eligibility to display a health claim at a score of <1, but a HSR 3.5 star rating corresponds to a score of ≤1. This relatively small difference accounted for 196/260 (75%) milk products that had a HSR of 3.5 or more, but were ineligible to display health claim.

Another reason for divergence between the two systems is because the NPSC scoring algorithm uses a dichotomous cut-point to determine eligibility to display health claims (eligible/not eligible). In contrast, the HSR system is a measure of the relative healthiness of foods from half a star to five stars in half-star increments i.e., a 10-point scale. In allocating star ratings to foods based on relative healthiness, it is inevitable that foods in the middle of the distribution may fall on either side of a dichotomous threshold for health claims. Nevertheless, notable anomalies in key categories where foods allocated HSRs of 3.0 or less could display health claims (oils, yoghurts, and soft drinks) indicate a need to review both systems in parallel with a view to achieving better alignment and minimizing consumer confusion.

A previous study that examined the nutritional profile of Australian and New Zealand foods found that 47% of Australian products were eligible to display a health or nutrient content claim under the NPSC, and also showed category level results in line with our current findings [15]. Only one previous study has evaluated the agreement between the NPSC and HSR. That study, which focused only on dairy products, also found good agreement between the NPSC and HSR ratings of >3 stars (Cohen’s kappa *k* = 0.78) [16].

Previous research has shown that the presence of health and nutrient content claims on product packaging can significantly influence consumer perception about the healthiness of foods [17], and that front-of-pack labels show promise in reducing bias associated with health claims. HSR labels, when compared to health claims and other types of front-of-pack labels, were found to have the lowest risk of creating an inaccurate positivity bias in unhealthy foods [18]. This again supports the need to ensure that HSR and NPSC are aligned consistently for all food categories, particularly those that may encompass less healthy food choices.

Importantly, this study is one of the first to calculate HSRs across the Australian packaged food supply. A similar New Zealand analysis showed that 36% of packaged food products would receive ≥3.5 stars, a lower proportion than we observed in the Australian food supply in this study (45%) [19]. However, there were differences in food categories included in two analyses that could explain this discrepancy. The HSR scheme, launched in June 2014 by the Australian government, has shown substantial uptake by manufacturers to date, with 7500 products in the FoodSwitch database now displaying a HSR on front-of-pack [10]. We observed that categories generally deemed as “healthy”, such as cereal products, fruit, vegetables, nuts and legumes, foods for special dietary use, and seafood, had a larger proportion of products with higher HSRs, and those deemed “unhealthy”, such as confectionery, had a larger proportion of products receiving a low HSR. This anticipated finding is positive, and along with the variation in HSRs observed within and between food categories, supports the notion that the HSR is a useful tool to help consumers identify healthier packaged food choices.

The findings of this study are subject to some limitations. The nutrition data used were as reported on food package labels, and we did not have information on which products actually displayed a health claim on-pack. It would be useful to conduct in-store surveys to determine alignment between actual HSR and health claims on-pack, in order to identify any instances of non-alignment between HSR ratings and health claims, and investigate the effects such occurrences have on consumer understanding of health, nutrition, and eventual food purchases. Future research in this area would also benefit from analysis of food sales data to determine how frequently anomalies between HSR and NPSC arise on frequently purchased foods.

## 5. Conclusions

We found good overall agreement between the HSR and the NPSC, with 97% products receiving a ≥3.5 HSR also eligible to carry health claim, and 94% products receiving <3.5 stars ineligible to display a health claim. However, anomalies were apparent in certain food categories, which could lead to consumer confusion. To minimize confusion, a detailed review should be undertaken of the alignment of scoring and performance of the two systems. Ensuring that products with low star ratings are not eligible to display health claims is critical to minimize confusion and retain consumer trust in food labelling.

## Figures and Tables

**Table 1:** Mean Health Star Ratings (HSR), proportion with HSR ≥3.5 and proportions eligible to carry health claim, overall and for major food groups.

Category	*n*	Mean HSR ± SD	No. (%) of Products with HSR ≥3.5	No. (%) of Products Eligible for Health Claim
Bread and bakery products	5125	2.3 ± 1.2	1472 (28.7%)	1549 (30.2%)
Cereal and cereal products	4511	3.6 ± 1	3150 (69.8%)	3182 (70.5%)
Confectionery	3681	1.3 ± 0.8	175 (4.75%)	252 (6.85%)
Convenience foods	2783	3.4 ± 0.6	1941 (69.7%)	2181 (78.4%)
Dairy	5515	2.9 ± 1.3	2200 (39.9%)	2292 (41.6%)
Edible oils	768	2.7 ± 1.3	342 (44.5%)	495 (64.5%)
Seafood and seafood products	1390	3.6 ± 0.8	1142 (82.2%)	1033 (74.3%)
Fruit, vegetables, nuts and legumes	4928	3.5 ± 1.1	3186 (64.7%)	3208 (65.1%)
Meat and meat products	2648	2.7 ± 1.3	1158 (43.7%)	948 (35.8%)
Non-alcoholic beverages	3485	2.7 ± 1.7	1336 (38.3%)	2003 (57.5%)
Sauces, dressings and spreads	4540	2.6 ± 1.3	1584 (34.9%)	1733 (38.2%)
Snack foods	1605	2.9 ± 1.2	694 (43.2%)	747 (46.5%)
Foods for special dietary use	318	3.6 ± 1.3	223 (70.1%)	208 (65.41%)
Total	41,297	2.8 ± 1.4	18,603 (45.1%)	19,831 (48.0%)

**Table 2:** Agreement between the Health Star Rating and the eligibility to display a health claim using the Nutrient Profiling Scoring Criterion.

Health Star Rating (HSR)	No. of Products with Each HSR (%)	No. of Products Eligible to Carry a Health Claim (%)	Sensitivity	Specificity	Kappa
0.5	3533 (8.6%)	2 (0.1%)	100.00%	0.00%	0.00
1.0	3412 (8.3%)	17 (0.5%)	99.99%	16.45%	0.16
1.5	4124 (10.0%)	29 (0.7%)	99.90%	32.26%	0.31
2.0	4433 (10.7%)	697 (15.7%)	99.76%	51.34%	0.50
2.5	3088 (7.5%)	245 (7.9%)	96.24%	68.75%	0.64
3.0	4104 (9.9%)	1368 (33.3%)	95.01%	81.99%	0.77
3.5	6298 (15.25%)	5405 (85.8%)	88.11%	94.74%	0.83
4.0	6375 (15.4%)	6168 (96.75%)	60.85%	98.90%	0.61
4.5	2886 (7.0%)	2864 (99.2%)	29.75%	99.86%	0.30
5.0	3044 (7.4%)	3036 (99.7%)	15.31%	99.96%	0.16
Total	41,297 (100%)	19,831 (48%)			

## Supplementary Materials

The following are available online at http://www.mdpi.com/2072-6643/10/8/1065/s1, Figure S1: Example calculations of the Health Star Rating and Nutrient Profiling Scoring Criterion, Figure S2: Distribution of Health Star Rating Score across food categories, Figure S3: Distribution of Nutrient Profile Scoring Criterion Score across food categories, Figure S4: Agreement between Health Star Rating Score and Nutrient Profiling Criterion Score by major food category, Table S1: Agreement between the number of products scoring ≥3.5 stars using the Health Star Rating and the proportion of products eligible to display a health claim using the Nutrient Profiling Scoring Criterion.
