# Supplementary material for: Evaluation of Alignment between the Health Claims Nutrient Profiling Scoring Criterion (NPSC) and the Health Star Rating (HSR) Nutrient Profiling Models
Source: Nutrients. 2018 Aug 10;10(8):1065. doi: 10.3390/nu10081065 (PMC6115993; doi:10.3390/nu10081065)
Supplement: Supplementary file 1 [file nutrients-10-01065-s001.pdf]

Table S1: Agreement between the number of products scoring >=3.5 stars using the Health Star Rating and the proportion of products eligible to display a health claim using the Nutrient Profiling Scoring Criterion

Category	No. of products <3.5 Health Star Rating eligible to display health claim (%)	No. of products >=3.5 Health Star Rating ineligible to display health claim (%)
**Bread and bakery products**	**92 (1.80%)**	**15 (0.29%)**
Biscuits	29 (1.31%)	7 (0.32%)
Bread	47 (3.21%)	4 (0.27%)
Cakes, muffins and pastries	16 (1.10%)	4 (0.28%)
**Cereal and cereal products**	**101 (2.24%)**	**69 (1.53%)**
Breakfast cereals	13 (0.92%)	25 (1.77%)
Cereal and nut-based bars	8 (1.13%)	21 (2.95%)
Couscous	0 (0.00%)	0 (0.00%)
Noodles	39 (8.99%)	0 (0.00%)
Pasta	26 (2.85%)	6 (0.66%)
Rice	9 (2.63%)	4 (1.17%)
Other cereal and grain products	6 (0.97%)	13 (2.10%)
**Confectionery**	**79 (2.15%)**	**2 (0.05%)**
Chocolate and sweets	12 (0.35%)	2 (0.06%)
Jelly	54 (39.42%)	0 (0.00%)
Chewing gum	3 (3.03%)	0 (0.00%)
Cough lollies	10 (32.26%)	0 (0.00%)
**Convenience foods**	**272 (9.77%)**	**32 (1.15%)**
Pizza	6 (3.59%)	7 (4.19%)
Pre-prepared salads and sandwiches	35 (7.64%)	1 (0.22%)
Ready meals	89 (7.02%)	24 (1.89%)
Soup	140 (16.11%)	0 (0.00%)
Other frozen foods not otherwise specified	2 (9.52%)	0 (0.00%)

**Dairy**	**409 (7.42%)**	**317 (5.75%)**
Cheese	9 (0.65%)	56 (4.07%)
Cream	2 (1.20%)	0 (0.00%)
Desserts	33 (7.97%)	0 (0.00%)
Ice cream and edible ices	29 (2.86%)	1 (0.10%)
Milk	1 (0.08%)	260 (21.21%)
Yoghurt and yoghurt drinks	335 (25.42%)	0 (0.00%)
**Edible oils**	**153 (19.92%)**	**0 (0.00%)**
Cooking oils	67 (18.72%)	0 (0.00%)
Edible oils	81 (24.40%)	0 (0.00%)
Cooking oil spray	5 (15.15%)	0 (0.00%)
Coconut oil	0 (0.00%)	0 (0.00%)
**Seafood and seafood products**	**10 (0.72%)**	**119 (8.56%)**
**Fruit, vegetables, nuts and legumes**	**257 (5.22%)**	**235 (4.77%)**
Fruit	179 (12.96%)	59 (4.27%)
Dried herbs and spices	9 (2.06%)	0 (0.00%)
Jam and marmalades	2 (0.50%)	3 (0.75%)
Nuts and seeds	6 (0.68%)	173 (19.70%)
Vegetables	61 (3.33%)	0 (0.00%)
**Meat and meat alternatives**	**31 (1.17%)**	**241 (9.10%)**
Meat alternatives	7 (3.10%)	3 (1.33%)
Processed meat	24 (0.99%)	238 (9.83%)
**Non-alcoholic beverages**	**678 (19.45%)**	**11 (0.32%)**
Coffee and tea	38 (16.59%)	4 (1.75%)
Cordials	85 (41.87%)	(0.00%)
Electrolyte drinks	11 (15.94%)	(0.00%)
Energy drinks	27 (21.26%)	(0.00%)
Fruit and vegetable juices	40 (2.74%)	0 (0.00%)
Soft drinks	299 (29.72%)	(0.00%)
Waters	163 (52.75%)	2 (0.65%)

Beverage mixes	15 (18.52%)	5 (6.17%)
**Sauces, dressings and spreads**	**208 (4.58%)**	**59 (1.30%)**
Mayonnaise and salad dressings	9 (1.65%)	0 (0.00%)
Sauces	130 (4.73%)	13 (0.47%)
Spreads and dips	69 (5.55%)	46 (3.70%)
**Snack foods**	**68 (4.24%)**	**15 (0.93%)**
**Foods for special dietary use**	**0 (0.00%)**	**15 (4.72%)**
Diet drink mixes (meal replacements)	0 (0.00%)	4 (2.67%)
Diet soup mixes (meal replacements)	0 (0.00%)	0 (0.00%)
Breakfast beverages	NA	11 (6.88%)
**Total**	**2358 (5.71%)**	**1130 (2.74%)**

Step 1: Determine the HSR category of the food

- *Category 1 = non-dairy beverages*
- *Category 1D = dairy beverages*
- *Category 2 = foods*
- *Category 2D = dairy foods*
- *Category 3 = fat, oils*
- *Category 3D = high fat cheeses*

Breakfast cereal product falls under category 2

Nutrient	Value per 100g
Energy (kJ)	1706
Protein (g)	9.1
Saturated fat (g)	2.5
Total sugars (g)	17.3
Sodium (mg)	20
Fibre (g)	6.8
FVNL%	0
Concentrated FVNL%	14

Step 2: Calculate baseline points (click here for lookup tables)

- *Energy points = 5*
- *Saturated fat points = 2*
- *Total sugars points = 3*
- *Sodium points = 0*

Baseline points = 5 + 2 + 3 + 0, which equals 10

Step 3: Calculate modifying points

- *Protein points = 5*
- *Fibre points = 7*
- *V points = 0*

Modifying points = 5 + 7 + 0, which equals 12

Step 4: Calculate final HSR score

Final score = Baseline points – Modifying points

= 10 – 12

= -2

Step 5: Calculate final HSR

An HSR score of -2 equals a HSR of 4

Figure S1: Example calculations of the Health Star Rating and Nutrient Profiling Scoring

(A) ***Calculation of the Health Star Rating – product example: breakfast cereal***

Step 1: Determine the NPSC category of the food

- *Category 1 = beverages*
- *Category 2 = foods*
- *Category 3 = fat, oils*

Breakfast cereal product falls under category 2

Step 2: Calculate baseline points (click here for lookup tables)

- *Energy points = 5*
- *Saturated fat points = 2*
- *Total sugars points = 3*
- *Sodium points = 0*

Baseline points = 5 + 2 + 3 + 0, which equals 10

Step 3: Calculate modifying points

- *Protein points = 5*
- *Fibre points = 5*
- *V points = 0*

Modifying points = 5 + 5 + 0, which equals 10

**Nutrient**	**Value per 100g**
Energy (kJ)	1706
Protein (g)	9.1
Saturated fat (g)	2.5
Total sugars (g)	17.3
Sodium (mg)	20
Fibre (g)	6.8
FVNL%	0
Concentrated FVNL%	14

Step 4: Calculate final NPSC score

Final score = Baseline points – Modifying points

= 10 – 10

= 0

Step 5: Determine eligibility

Requires score <4 to be eligible

Product is eligible to display a health claim

Figure S1: Example calculations of the Health Star Rating and Nutrient Profiling Scoring

(B) ***Calculation of the Nutrient Profiling Scoring Criterion – product example: breakfast cereal***

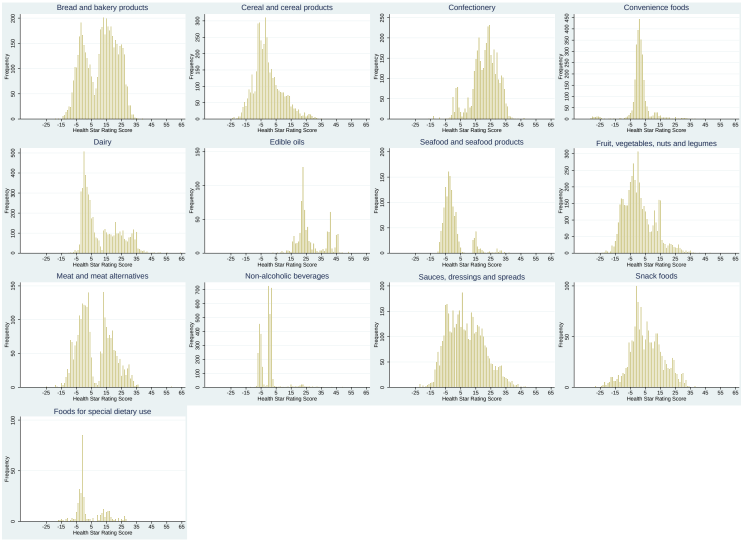
Bread and bakery products
Cereal and cereal products
Confectionery
Convenience foods
Dairy
Edible oils
Seafood and seafood products
Fruit, vegetables, nuts and legumes
Meat and meat alternatives
Non-alcoholic beverages
Sauces, dressings and spreads
Snack foods
Foods for special dietary use
Frequency
Health Star Rating Score
-25 -15 -5 5 15 25 35 45 55 65

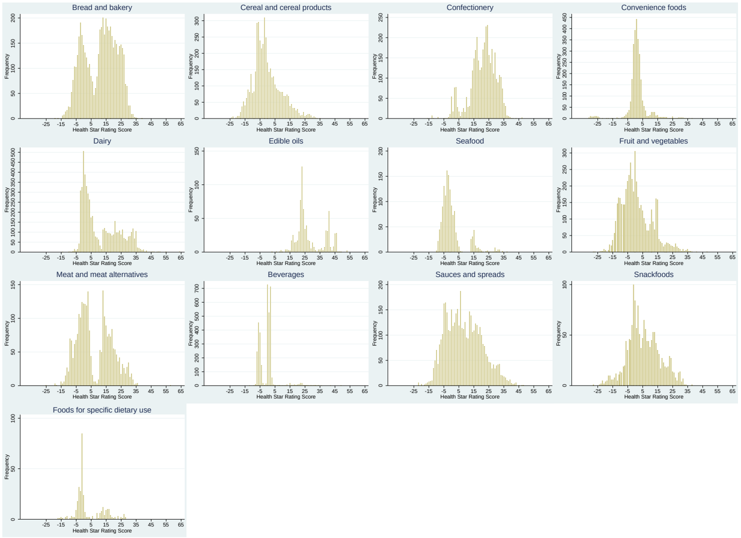

<u>Figure S2:</u> Distribution of Health Star Rating Score across food categories

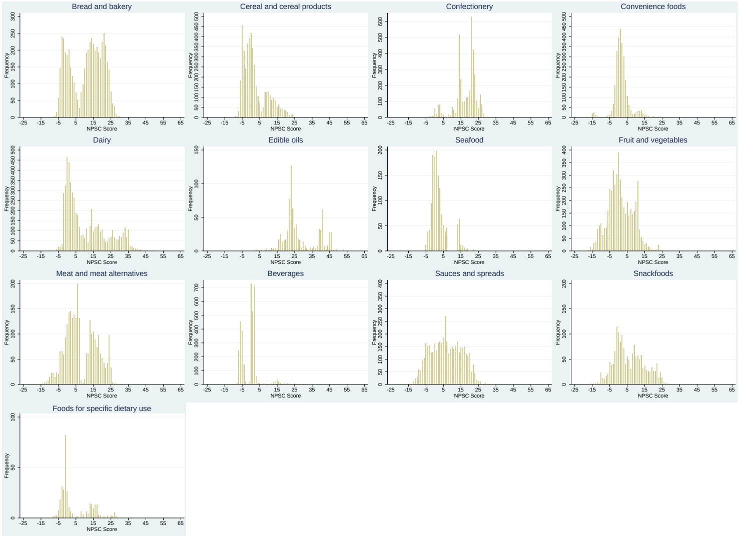
Bread and bakery
Cereal and cereal products
Confectionery
Convenience foods
Dairy
Edible oils
Seafood
Fruit and vegetables
Meat and meat alternatives
Beverages
Sauces and spreads
Snackfoods
Foods for specific dietary use
Frequency
NPSC Score

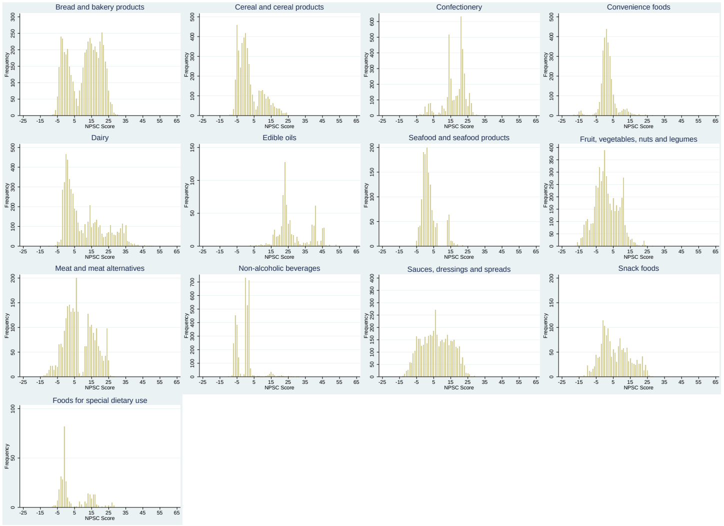

<u>Figure S3:</u> Distribution of Nutrient Profile Scoring Criterion Score across food categories

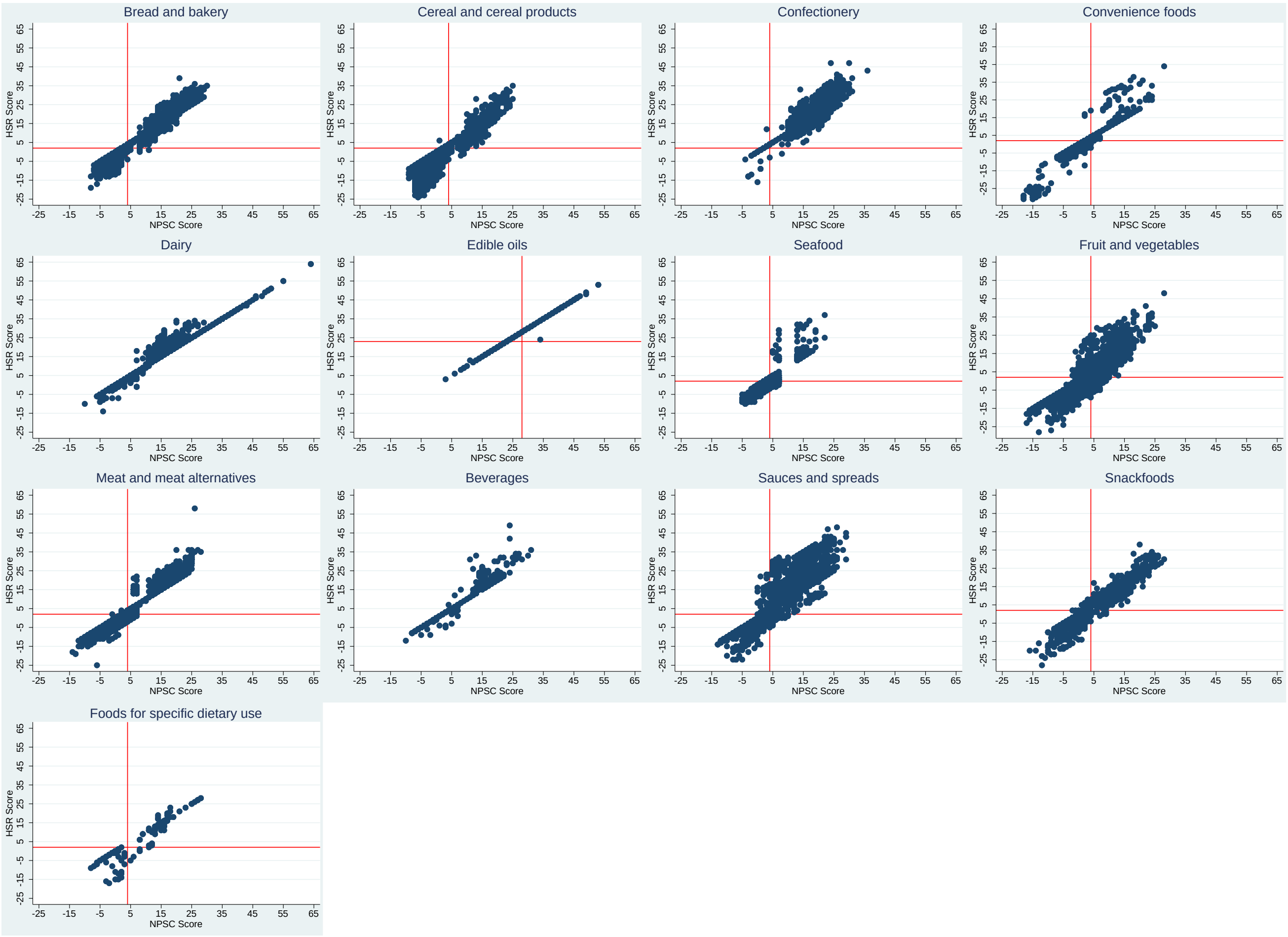
Bread and bakery
Cereal and cereal products
Confectionery
Convenience foods
Dairy
Edible oils
Seafood
Fruit and vegetables
Meat and meat alternatives
Beverages
Sauces and spreads
Snackfoods
Foods for specific dietary use
HSR Score
NPSC Score
-25 -15 -5 5 15 25 35 45 55 65

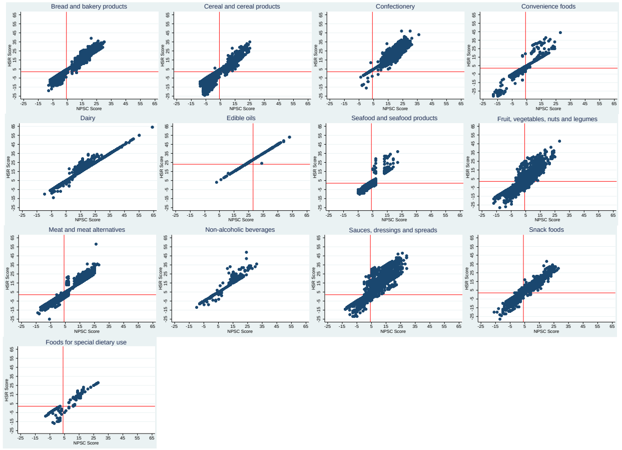

Figure S4: Agreement between Health Star Rating Score and Nutrient Profiling Criterion Score (NPSC) by major food category

The horizontal red line indicates the cut-off value to have a HSR star of 3.5 and the vertical red line indicates the cut-off for eligibility to display a health claim under the NPSC. The bottom left and top right corners indicate agreement between the HSR and NPSC and the top left and bottom right indicate disagreement.

Beverages and Dairy categories contained a mixture of NPSC and HSR classifications and so do not include a cut-off line.
